# Polyphenolic Compounds and Digestive Enzymes: In Vitro Non-Covalent Interactions

**DOI:** 10.3390/molecules22040669

**Published:** 2017-04-22

**Authors:** Alejandra I. Martinez-Gonzalez, Ángel G. Díaz-Sánchez, Laura A. de la Rosa, Claudia L. Vargas-Requena, Ismael Bustos-Jaimes, Emilio Alvarez-Parrilla

**Affiliations:** 1 Departamento de Ciencias Químico Biológicas, Instituto de Ciencias Biomédicas, Universidad Autónoma de Ciudad Juárez, Ciudad Juárez 32310, Mexico; aimg.2086@gmail.com (A.I.M-G.); angel.diaz@uacj.mx (Á.G.D.-S.); ldelaros@uacj.mx (L.A.d.l.R.); cvargas@uacj.mx (C.L.V.-R.); 2 Departamento de Bioquímica, Facultad de Medicina, Universidad Nacional Autónoma de México, México D.F. 04510, Mexico; ismaelb@unam.mx

**Keywords:** polyphenolic compounds, structure, digestive enzymes, enzymatic inhibition, van der Waals forces, hydrogen binding, hydrophobic binding

## Abstract

The digestive enzymes–polyphenolic compounds (PCs) interactions behind the inhibition of these enzymes have not been completely studied. The existing studies have mainly analyzed polyphenolic extracts and reported inhibition percentages of catalytic activities determined by UV-Vis spectroscopy techniques. Recently, pure PCs and new methods such as isothermal titration calorimetry and circular dichroism have been applied to describe these interactions. The present review focuses on PCs structural characteristics behind the inhibition of digestive enzymes, and progress of the used methods. Some characteristics such as molecular weight, number and position of substitution, and glycosylation of flavonoids seem to be related to the inhibitory effect of PCs; also, this effect seems to be different for carbohydrate-hydrolyzing enzymes and proteases. The digestive enzyme–PCs molecular interactions have shown that non-covalent binding, mostly by van der Waals forces, hydrogen binding, hydrophobic binding, and other electrostatic forces regulate them. These interactions were mainly associated to non-competitive type inhibitions of the enzymatic activities. The present review emphasizes on the digestive enzymes such as α-glycosidase (AG), α-amylase (PA), lipase (PL), pepsin (PE), trypsin (TP), and chymotrypsin (CT). Existing studies conducted in vitro allow one to elucidate the characteristics of the structure–function relationships, where differences between the structures of PCs might be the reason for different in vivo effects.

## 1. Introduction

**Figure 1:**
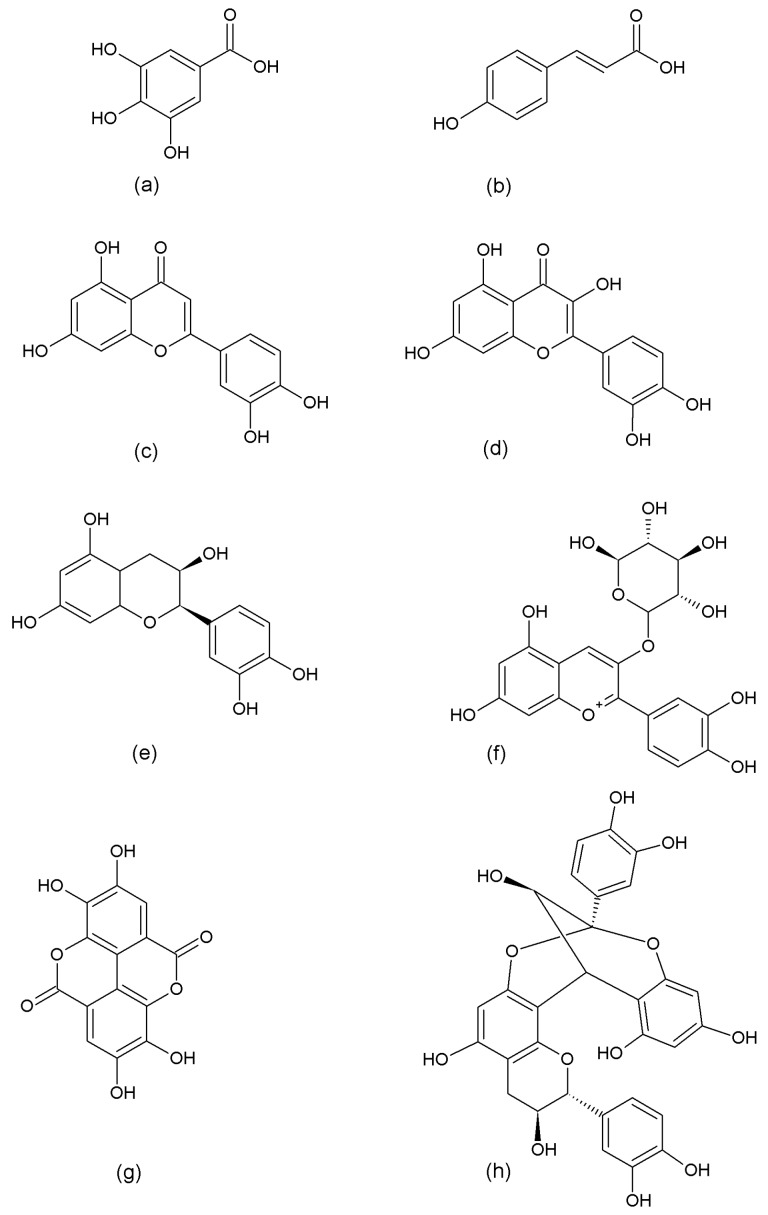
The chemical structures of some representative polyphenolic compounds examples, (**a**) gallic acid; (**b**) *p*-coumaric acid; (**c**) luteolin (**d**) quercetin; (**e**) (−)-epicatechin; (**f**) cyanidin-3-*o*-glucoside; (**g**) ellagic acid; and (**h**) proanthocyanidin A1.

Polyphenolic compounds (PCs) are plant secondary metabolites that are involved in functions such as defense against predators, protection against UV light damage and environmental stress or reproduction, among others [1]. In general, PCs can be classified as phenolic acids, flavonoids, tannins, stilbenes, curcuminoids, lignans, and others [1,2,3]. The largest groups of PCs are the phenolic acids, flavonoids and tannins (see Figure 1 for examples). Phenolic acids, which possess one aromatic ring and at least one carboxylic acid moiety in their structure, are divided into two groups, hydroxybenzoic acids (C6-C1) (i.e., gallic acid) and hydroxycinnamic acids (C6-C3) (i.e., *p*-coumaric acid). The flavonoids exhibit a three ring structure base (C6-C3-C6), and are divided in several subgroups such as flavones, flavonols, flavanones, flavanols and others [4], depending on the substitutions in the central heterocyclic ring, while tannins are polymeric PCs with higher molecular weights, that can be divided into two subgroups, hydrolyzable tannins and condensed tannins, such as ellagitannins and proanthocyanidins, respectively [5]. 

Some of the recognized properties of PCs include antioxidant [6], antiproliferative [7], antibacterial [8], antiviral [9], anti-inflammatory [1], antidiabetic [10] anti-obesity activity [11], and others. However, the antidiabetic and anti-obesity activities related to digestive enzymes are the focus of this work. The antidiabetic activity of PCs is mostly related to their effectiveness in diminishing post-prandial glycemic levels, i.e., as described by da Silva et al. [12]. This effect can be attributed to the PCs reducing the absorption of glucose by inhibition of digestive enzymes. Several mechanisms may account for the anti-obesity activity of PCs. They modulate the expression of genes that control lipid metabolism [13], thermogenesis and energetic expenditure [14], but their benefit on human health is mainly associated to the binding of PCs to macromolecules such as enzymes [15,16]. In this scenario, specific binding of dietary PCs with enzymes of the digestive tract that are responsible for the lipid absorption in the small intestine may be considered one of the main mechanisms by which PCs can prevent obesity [11,17,18,19,20,21]. It has been suggested that the inhibition of digestive enzymes could decrease postprandial hyperlipidemia and obesity [22]. 

The use of synthetic or natural drugs that inhibit digestive enzymes has been proposed as a treatment for obesity. Orlistat was the first lipase inhibitor approved as an anti-obesity drug by the U.S. Food and Drug Administration in 1999 [23]. This drug is a synthetic hydrogenated derivative of a lipostatin produced by *Streptomyces toxytricini*. Other lipase inhibitors such, as lorcaserin, were accepted for obesity treatment. There have been reported undesired side effects from these drugs intake, including mild symptoms such as oily spotting and severe evacuations [23], or even more severe effects such as myocardial infarction [24]. Similarly, the inhibition of the enzymes responsible for the carbohydrate absorption are mainly studied for the control of postprandial hyperglycemia in diabetes and also obesity [25]. Acarbose, a carbohydrate-hydrolyzing enzyme inhibitor that naturally occurs in *Streptomyces* sp. produces undesirable gastrointestinal disturbances [26]. Due to the side effects of these drugs over the digestive metabolism, new alternatives have been evaluated, among them PCs, as potential therapeutic agents for obesity and diabetes, acting as enzymes inhibitors [27]. 

It should be noted that digestion of proteins is also part of the full digestive system, and some PCs are also known to inhibit protein absorption. This has been mainly described for high concentrations of tannins that nonspecifically bind and precipitate proteins and therefore, are considered as anti-nutritional compounds [28]. However, only few authors like Xiao et al. [29] have analyzed the inhibition of digestive proteases such as trypsin, by PCs. Since no disease as obesity or diabetes has been associated to polypeptide absorption, inhibition of digestive proteases would be an undesired side effect of PCs and should also be evaluated when searching for inhibition of lipases and carbohydrate-hydrolyzing enzymes.

Considering the diverse structure of PCs, studies on the structure–activity relationship (SAR) of PCs and their digestive enzyme inhibition activity could help to understand the structural features of PCs that are most important for this activity and propose an inhibition mechanism [26]. Furthermore, this information can be the basis for developing new and more effective anti-obesity and antidiabetic agents, for which, according to Buchholz and Melzig [4], “The innovative approach lies in using the structure of a known potent inhibitor”. The SAR of PCs has been reviewed, i.e., related to their bioavailability and bioactivity [30]. The aim of this review is to describe the principal findings regarding the interactions of PCs and some digestive enzymes, by discussing structural differences of the analyzed PCs and their subsequent effect on digestive enzymes activities. In addition, the main techniques used for these interactions analysis are described.

## 2. Results

### 2.1. Digestive Enzymes

**Figure 2:**
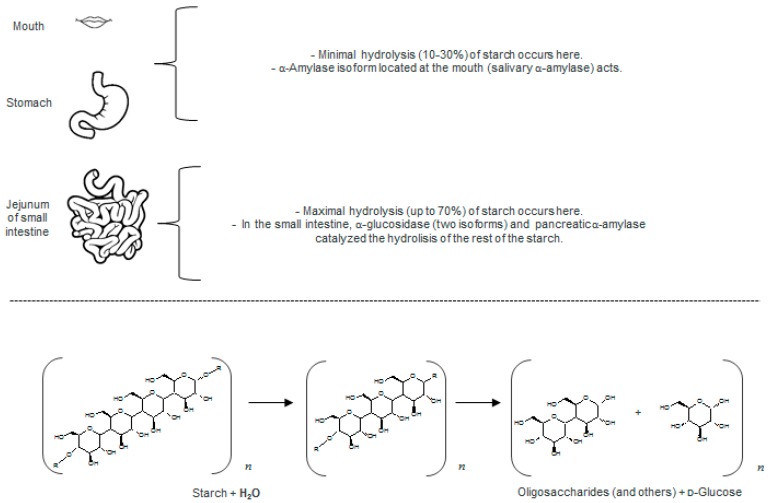
Example of digestive enzymatic activity. An abstract of main carbohydrate-hydrolyzing enzymes, α-glucosidase and α-amylases isoforms, over starch is presented.

Both nutrients and non-nutrients present in foods matrices are released by the digestive process in humans [31]. This digestive process can be divided into three stages: salivary, gastric and intestinal digestion [32]. Each stage is a complex process that involves the presence of enzymes such as carbohydrate-hydrolyzing enzymes, lipases and proteases, bile salts, and particular pH conditions [33]. Up to 70% of the hydrolysis of dietary macromolecules that serve as nutrients (carbohydrates, lipids and polypeptides) occurs in the intestinal stage [17,34]. Figure 2 schematizes the role of the enzymes responsible for the breakdown of dietary starch (the carbohydrate-hydrolyzing enzymes, α-glucosidase (EC 3.2.1.20) and α-amylase (EC 3.2.1.1)), prior to their absorption of oligo and monosaccharides.

#### 2.1.1. Glucosidase and Amylase Enzymes

The main sources of glucose in humans are the complex carbohydrates starch and glycogen. The action of carbohydrate-hydrolyzing enzymes in the organism is the hydrolysis of α-glycosidic links in polysaccharides, to produce glucose and small oligosaccharides that can be absorbed in the small intestine (Figure 2). In humans two α-amylase isoforms have been reported, salivary and pancreatic [35], whereas two α-glucosidase isoforms are located at small intestine [36]. Each α-glucosidase isoform possesses an activity, one is a maltase-glucoamylase (MGAM) and the other is a sucrose-isomaltase (SI). Lin et al. [36] broadly described each isoform activity, where amino-terminal and carboxy-terminal subunits have different activities; i.e., the amino-terminal subunit of MGAM acts as maltase, and its carboxy-terminal subunit has a glucoamylase activity. Since the amino-terminal subunit of each enzyme possesses the catalytic site, and the MGAM enzyme has a higher hydrolytic activity than the SI isoform, then the amino-terminal subunit of MGAM isoform is mentioned as the main α-glucosidase [37,38]. In this way, the denomination “α-glucosidase” refers to maltase-glucoamylase isoform (EC 3.2.1.20). Figure 2 schematizes the hydrolysis of carbohydrates, which begins in the mouth with the action of the salivary α-amylase. Its action, however, is limited for example by the short time that food remains in the mouth, thus pancreatic isoform activity in the digestive tract shows the highest rate with more than 70% of the overall hydrolysis [19,26,37].

**Figure 3:**
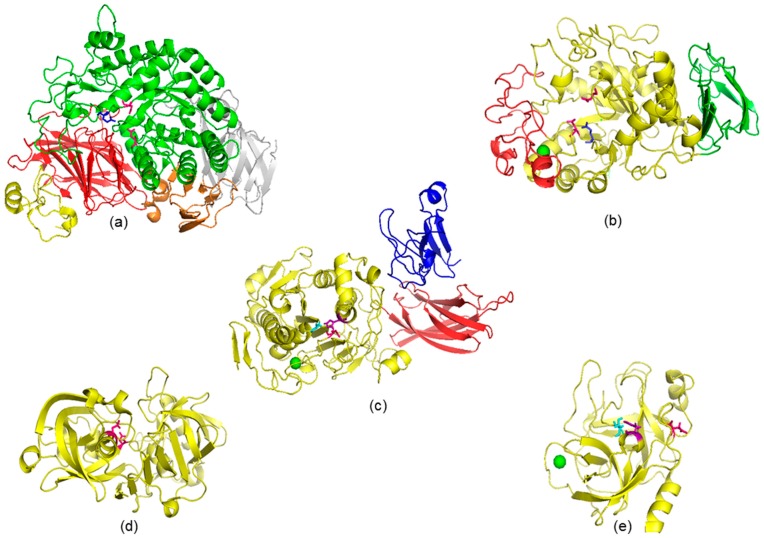
Three-dimensional structures of digestive enzymes: (**a**) α-glucosidase (PDB accession No.: 2QLY); (**b**) pancreatic α-amylase (No.: 1PIF2); (**c**) pancreatic lipase (No.: 1ETH); (**d**) pepsin (No.: 1YX9) and (**e**) trypsin (No.: 1S81). Domains A, B and C are presented in yellow, red and green colors, respectively; while domains D and E of α-glucosidase are presented in orange and gray colors, respectively. Colipase in pancreatic lipase is presented in blue color. The amino acid residues from the active site of each enzyme are colored: pink for Asp, blue for Glu, aquamarine for Ser, and purple for His. Ca^2+^ ion is a green-colored dot.

According to Bhandari et al. [37] and Miao et al. [19] among the carbohydrate-hydrolyzing enzymes, α-amylase (approx. 55 kDa) and α-glucosidase (260 kDa) located at small intestine are two of the main carbohydrate hydrolases. α-Amylase hydrolyzes α-1,4 internal linkages of starch into maltose, maltotriose and limit dextrins with an average size of eight glucose units [39]; whereas α-glucosidase hydrolyzes terminal, non-reducing α-D-1,4 linkages releasing glucose and oligosaccharides [40]. They are pancreatic exocrine enzymes. α-Glucosidase (abbreviated as AG, Figure 3a) can be divided into five domains (A, B, C, D and E; yellow, red, green, orange and gray), while α-amylase (PA, Figure 3b) present in their structure only three domains (A, B and C) [41,42]. α-Glucosidase and α-amylase are 868- and 496-amino acid proteins, respectively. The catalytic triad is located inside the (β/α)_8_ barrel domain, C domain for AG and A domain for PA. The catalytic triad of PA is composed by Asp^197^, Glu^223^ and Asp^300^ [41,43].The catalytic triad of AG is not clear enough, but the amino acid residues Asp^443^ and Asp^542^ can be part of its catalytic site [38,42], as well as Glu^444^, if we analyzed the conformation of catalytic domain (270–651 amino acid residues) and the presence of a Glu residue in other α-glucosidase. Crystallographic studies have shown that PA domain C folds into an eight-stranded antiparallel β-barrel, while domain B is formed by antiparallel β-sheets [44]. Also, PA enzyme structure binds one Ca^2+^ ion, which facilitates the bind between the A and B domains [45]. While AG domain A presents a trefoil Type-P, and the others non-catalytic domains (B, D and E) present β-sandwich structures [38]. 

The activity of these two enzymes can be localized in the brush borders of the jejunum enterocytes, where the hydrolytic activity of α-amylase is followed by that of α-glucosidase. Recently, Lin et al. [36] discussed the synergistic effect of α-amylase and α-glucosidase, they mentioned that the AG action does not necessary follow the PA action. These two enzymes can be acting at the same time, whereas the AG activity would be the rate-limiting step in starch hydrolysis. In this way, the activity of AG would attract more interest than it has, on the inhibition studies of digestive enzymes.

#### 2.1.2. Lipase Enzyme

Dietary triacylglycerides are hydrolyzed by enzymes called lipases. In humans there are pre-duodenal and extra-duodenal lipases [46]. The pre-duodenal lipases isoforms include lingual and gastric; and the extra-duodenal lipases include pancreatic lipase (EC 3.1.13), Figure 3c, abbreviated as PL), which is responsible for the hydrolysis ca. 70% of total dietary lipids [17]. The activity of this extra-duodenal enzyme, PL (50 kDa), in the small intestine is essential for the dietary lipids digestion. PL releases fatty acids from sn1 and/or sn3 position of dietary triacylglycerides, yielding monoglycerides, diglycerides and free fatty acids as products of the lipolytic reaction [47]. According to Miled et al. [48], PL is a 449-amino acid protein divided into two domains (amino-terminal and carboxy-terminal). The amino-terminal domain presents an α/β hydrolase fold, which possess the catalytic triad (Ser^152^, Asp^176^ and His^263^). The carboxy-terminal domain, which is a β-sandwich, interacts by non-covalent bonds with its cofactor (colipase), necessary for its enzymatic activity. Whereas PL presents a form called closed in absence of colipase. A PL open form is induced by contact with the lipid-water interface (substrate) in presence of colipase [49,50]. PL Lid domain and β5 loop, which are covering its active site (hydrophobic β9 loop), will undergo conformational changes. Then the colipase founds its own binding site from the new arrangement of the Lid domain. Also, a Ca^2+^ ion located far away from the catalytic site plays a role in PL structure. Lipolysis requires the presence of PL, colipase and bile salts [49,51]. These authors observed by crystallographic studies, that colipase only binds to the non-catalytic domain of PL.

#### 2.1.3. Proteases Enzymes

Dietary polypeptides are converted into smaller polypeptides and amino acids by digestive proteases. Pepsin ((EC 3.4.23.1), abbreviated as PE), chymotrypsin ((EC 3.4.21.4), CT) and trypsin ((EC 3.4.21.4), TP) are proteases of the digestive system. These enzymes have mainly β-sheets structures. Ibarz et al. [52] explained that while pepsin is produced as zymogen by the gastric chief cells in the stomach wall, chymotrypsin and trypsin are synthesized by the acinar cells of pancreas as zymogens. Later, these zymogens get active in the small intestine by an irreversible covalent modification, which involves proteolytic cleavage of one of more peptidic bonds, i.e., an intestinal enteropeptidase hydrolyzes the peptidic bond between Lys^6^ and Ile^7^ of trypsinogen to produce active trypsin. 

Pepsin (Figure 3d) has a sequence of 385 amino acids, a molecular weight around 41 kDa, and a catalytic site formed by two Asp at positions 32 and 315 [53]. While trypsin (Figure 3e) and chymotrypsin are serine proteases, bound to a Ca^2+^ ion, with a catalytic triad of His^57^, Asp^102^ and Ser^195^. Even though the amino acid at position 189 does not belong to catalytic triad, it represents an important difference as part of the primary substrate-binding pocket of these two proteases. Trypsin possesses a negatively charged Asp at position 189, and chymotrypsin has a polar amino acid residue, Ser, at the same position [54]. The negative charge of Asp^189^ carboxylate in TP allows the interaction with the positive charge groups of Lys and Arg. TP contains 231 amino acids and has a molecular weight of 24 kDa; whereas CT has 266 amino acids and a molecular weight of 28 kDa. Each enzyme is responsible for the hydrolysis of different peptidic bonds. TP hydrolyzes the peptidic and ester bonds formed by the carboxyl group of the basic amino acids, Arg and Lys and any other residue [55]. CT hydrolyzes the peptide bonds formed by the carboxyl group of the aromatic or large hydrophobic amino acids, Phe, Tyr, Trp and Met and any other residue [56]; while PE exhibits preferential cleavage for aromatic residues in either positions of the peptide bond. 

### 2.2. Inhibition of Digestive Enzymes by Polyphenolic Compounds

Table 1 summarizes the published research studies on the inhibition of digestive enzymes by PCs. This table shows that most of the published studies report results as % Inhibition and IC_50_ values. Also these studies mainly analyze PCs extracts instead of pure PCs [57], and those studies regarding pure PCs compounds, reported mainly % inhibition values. Only few authors determined kinetic and affinity constants. It must be mentioned that only enzymes from porcine sources were evaluated, due to the structural homology between human and porcine; for example between human and porcine pancreatic lipase there is an 86% of structural homology [17].

The studies related to PCs and digestive enzymes inhibition have been carried out mainly with extracts. Table 1 exhibits that most of the studies have been carried out using green tea (*Camellia sinensis*) PCs extracts, fruits such as muscadine grapes (*Vitis rotundifolia*) or spices such as cumin (*Cuminum cyminum* L.). Tea extracts (green, black, oolong) have been largely studied as digestive enzyme inhibitors, probably due to their high consumption around the world [25]. It has been reported that tea extracts show different health beneficial properties such as antioxidant [58], lowering cholesterol levels, improving the immune system function [59], as well as the digestive system function [25]. Several concentrations of tea PCs extracts have been analyzed to inhibit PA activity, interestingly non-inhibitory results were observed at a concentration of 1.5 mg/mL [25], while a moderate to large PA inhibition was observed for guava (*Psidium guajava* L.) PCs extracts at the same concentration [60], indicating that not all PCs extracts are equally effective for inhibiting PA [61]. Several studies have reported PL inhibition by herbal or fruit PCs extracts [17,18,22], showing sometimes contradictory results. For example, Worsztynowicz et al. [62] didn’t observe any PL inhibition when a black chokeberry extract was used. In contrast, Podsedek et al. [63] analyzed the anti-lipase activity of crude extracts from thirty fruits, and they observed the lowest IC_50_ (higher inhibition) value for chokeberry extract. According to [25,62] other components of the extract might affect the activity of the enzymes, PA and PL, which could help explain the differences. In comparison with PA and PL, proteases (TP and CT) have been barely studied, using only tea PCs extracts [28,64] or pure flavonoids as inhibitors [65,66].

When authors identified the polyphenolic components of the extracts, the intromission of other components could be eliminating and the studies of the polyphenolic structures carrying out. Zhang et al. [21] analyzed the difference between flavonol glycosides and their aglycones. These authors studied the different effects of kaempferol and kaempferol-glucose, and quercetin and quercetin-arabinoside from lentil (*Lens cultivars*) cultivars extracts against pancreatic lipase activity, and they did not find statistically different values for each flavonoid pair. In a similar study, Li et al. [83] evaluated the effect of a glucoside substituent in position 3 of the C ring of quercetin, analyzing quercetin, isoquercetin and rutin. They observed that rutin, which possesses a disaccharide moiety was the best PL inhibitor, followed by isoquercetin (monosaccharide moiety) and finally quercetin (aglycone). These authors found that the placement of a double glycosylation, rutinoside for rutin or arabinoside for quercetin-arabinoside, respectively, provided a higher possibility to interact with the enzyme, by increasing the polarity of the PCs–protein adduct, by hydrogen bonding formation, and decreasing the hydrophobic environment near the catalytic site, necessary to hydrolyze the triacylglyceride [21,83]. Similar results (lower IC_50_ values for the glycoside) were observed by Miao et al. [19] when analyzing the inhibition of PA by resveratrol-3-*o*-glucoside from skin grape extracts. In this case, the authors suggested that glycosylation of the flavonoid may be interrupting the enzyme–substrate contact by binding to the substrate. Differences in the inhibitory activity of flavonoid glycosides due to the structure of the glycosyl moiety were also observed. According to Akkarachiyasit et al. [90] cyanidin-3-galactoside and cyanidin-3-glucoside, with a difference on C4 carbohydrate moiety, showed different inhibitory activity against PA. Dalar and Konczak [58] attributed the inhibitory activity of a *Plantago lanceolata* L. extract to the presence of luteolin-7-*o*-glucoside, indicating that other C-ring substitution positions (C5 and C7) may also be effective. 

For trypsin, the effect of increasing the number of hydroxyl groups were tested by Li et al. [65]. Four flavonoids—quercetin, luteolin, kaempferol and apigenin—were studied against trypsin activity, and the results suggested that the binding of flavonoids to trypsin increased with an increase in the number of hydroxyl groups (quercetin > luteolin > kaempferol > apigenin). Similar results have been reported by Kanakis et al. [91] and Jia et al. [92] for the interaction between catechins and β-lactoglobulin, where the binding constants of the PCs-β-lactoglobulin complexes with PCs that contained more hydroxyl groups were higher than those from PCs–protein complexes formed with smaller PCs. A larger number of hydroxyl groups in a PCs represented an advantage for binding and diminishing the enzymatic activity.

The higher inhibitory activity of flavonoids compared to phenolic acids, seems to be related with the complexity of the structures of the flavonoids when interacting with the enzyme. Sergent et al. [34] observed lower IC_50_ values (higher inhibitory effect) over PL for quercetin and kaempferol, compared to ferulic acid, or even Orlistat. You et al. [67] observed higher inhibitory effect of quercetin than ellagic acid over both AG and PL. Tan et al. [79] observed higher inhibition (lower IC_50_ value) of AG, PA and PL activities from the flavonoid myricetin than phenolic acids such as gallic acid, caffeic acid, sinapic acid, and others. Hu et al. [81] observed that the smaller caffeoylquinic acid derivatives also exhibited fewer or weaker binding to PL. A lower inhibition of PA activity was produced by chlorogenic acid derivatives from green coffee (*Coffea arabica*) extracts [69], than by flavonoids as quercetin, kaempferol and myricetin from guava leaves [60]. Even though flavonoids higher inhibitory activities than phenolic acid had been stablished, some authors have studied differences among inhibitory activities of chlorogenic acid and its derivatives from green coffee beans extract to inhibit PA [69]. Among their conclusions, they indicated that, as in flavonoids, the inhibition of PA also increased as the number of hydroxyl groups on the structure increased, which is attributed to the steric hindrance from hydroxyl groups. They also suggested that there could exist some structural characteristics that would affect the inhibitory effect, for example the neighboring two hydroxyl groups on the catechol ring, while others, such as a change of the hydroxyl group position from *p*-coumaric to *m*-coumaric acids, would not. In general terms, a more complex structure of PCs seems to be related with a higher affinity for enzymes. The comparison between phenolic acids and flavonoids related to protease activity has not been studied so far; however, by comparing the trypsin-inhibitory activity of quercetin and tannic acid in one study, we observe that the inhibition values of tannic acid [29] were higher than those of quercetin [65].

The inhibitory effect of individual PCs also varies depending of the enzyme analyzed. A higher effect of PCs over the activities of carbohydrate-hydrolyzing enzymes, AG and PA, compared to PL has been reported [74]. You et al. [67] observed a six-fold difference between quercetin IC_50_ value for AG compared to PL. Vazquez-Flores et al. [93] observed higher carbohydrate hydrolysis inhibition attributed to PA activity, in comparison with lipid hydrolysis inhibition attributed to PL activity (approx. 46 and 30% inhibition, respectively) when a proanthocyanidins fraction from pecan (*Carya illinoinensis*) extract was evaluated. Worsztynowicz et al. [62] observed that chlorogenic acid inhibited PA, while no inhibition over PL activity was detected. He et al. [28] studied the inhibition of PA, PL, TP and PE by tea PCs extracts (61, 54, 38 and 32% inhibition, respectively). These authors suggested that the higher inhibition of PA activity was attributed to its higher molecular weight compared to the other enzymes. These results are in agreement with Xiao et al. [29], who observed higher inhibition of AG (higher molecular weight) compared to TP, in the presence of tannic acid. However, more studies are needed to prove this hypothesis because He et al. [28] also reported that TP, which is smaller than PE, was inhibited in a higher degree by tea PCs extracts. Other enzyme properties such as protein polarity and conformational structure may also play an important role in enzyme inhibition [91,94]. However further studies about inhibition of these different hydrolases (AG, PA and PL) need to be done, because a few authors reported that some pure PCs like gallic acid and catechin [79], or different quinoa (*Chenopodium quinoa*) grain extracts [72] were better inhibitors of PL, followed by AG, and finally PA.

### 2.3. Structural and Thermodynamic Characterization of Polyphenolic Compounds–Protein Interactions

Polyphenolic Compounds–protein interactions have been analyzed mainly using milk proteins such as albumins, caseins and lactoglobulins as models, observing that PCs bind to the proteins by means of non-covalent interactions [16]. Hydrogen bonding has been reported as the main force in the interaction between chlorogenic acid and bovine serum albumin (BSA), α-lactalbumin and lysozyme, without pronounced effects on the functional properties of each protein [95]. Interactions between tea extracts with casein [96] and β-lactoglobulin [91] have been studied. Hydrophobic bonds between casein and green tea flavonoids and other PCs such as catechin, *p*-coumaric acid and gallic acid have been reported for β-lactoglobulin. Hydrophobic and hydrophilic bonds between catechins and β-lactoglobulin were detected [91]. It has been reported that the affinity between PCs and protein, may increase as the number of hydroxyl groups increases in the PCs structure [64] or as the number of hydrophilic sites on the protein increases [69].

To gain information regarding the type of interaction present between PCs and digestive enzymes, other techniques such as fluorescence spectroscopy (FLU) and calorimetric studies (isothermal titration calorimetry and differential scanning calorimetry) have been used (Table 1). The decrease in the intrinsic fluorescence intensity of a protein (quenching) has been used to study the affinity between PCs and digestive enzymes [11,64,67,83,97]. According to Lakowicz [98], two types of quenching processes between PCs and proteins may exist. The collisional quenching, is produced when the quencher (PCs) diffuses to the fluorophore during the lifetime of the excited state, then the fluorophore returns to the ground state, without emission of a photon. While the static quenching refers to the formation of a ground state complex between the fluorophore and quencher, resulting in a non-fluorescent complex. The fluorescence intensity quenching analysis can be used to determinate binding characteristics, between PCs (quenchers) and enzymes (fluorophores), because for either type of quenching, these two molecules must be in close contact. It has to be pointed out that the intrinsic fluorescence of protein measured by quenching is mainly contributed by its Trp residues [98]. Most of the reported studies described the PCs–digestive enzymes interactions using the static quenching approach. Gonçalves et al. [99] reported static quenching between grape seed procyanidins and PA. While Li et al. [83] in another study described the interactions between flavonoids (rutin, isoquercetin and quercetin) and PL also by static quenching. Both quenching mechanisms have been observed between sorghum (*Sorghum bicolor*) procyanidin trimers and PA [68]. Gonçalves et al. [99] observed higher affinity constants between PA and grape procyanidins, as their polymerization degree increased. Similarly, Li et al. [65] observed higher affinity values with higher molecular weight PCs, and the sequence of inhibition against TP activity was quercetin, luteolin, kaempferol and finally apigenin. The opposite effect was observed by Cai et al. [68] when analyzing the interaction between higher molecular weight sorghum procyanidins and PA, and the authors assumed that the steric hindrance of the higher molecular weight explained these differences. Considering that procyanidins are complex polymeric compounds, these results may be explained regarding not only the polymerization degree, but also the monomeric units of the procyanidin ((+)-catechin, (−)-epicatechin, (−)-gallocatechin and (−)-epigallocatechin), as well as the type of linkage present between monomers. However, more studies are required to elucidate the structure–activity relationship, especially in the case of complex polymers.

Fluorescence quenching data is adjusted to the Stern–Volmer equation, where the number of binding sites per protein (*n*) and the affinity constant (*K*_a_) are mainly calculated. Stern–Volmer quenching constant (*K*_sv_) and quenching rate constant (*k*_q_) can be calculated too. These parameters are calculated with the equation and linear regression of plots of fluorescence intensities in the absence and presence of quencher versus quencher concentration [98]. A *n* value of 1 (approximately) has been calculated for the interactions between digestive enzymes and PCs; for example: PA and sorghum procyanidins [68], PL and flavonoids [83], TP and flavonoids [65], CT and acteoside [64]. This indicates that there was only one binding site in enzyme for the PCs, and that a static quenching mechanism occurred on that site. Since the authors have observed a static quenching mechanism between digestive enzymes and PCs, they have used *K*_a_ as the static quenching constant [64,83]. Then, the *K*_sv_ is commonly named as the collisional quenching constant, because if the measurement of *K*_sv_ at different temperatures reveals that the increase of *K*_sv_ values is directly correlated with temperature, it suggests the occurrence of a collisional quenching mechanism. Skrt et al. [100] associated a higher *K*_sv_ value to a better inhibitor ((−)-epicatechin-3-gallate) of BSA activity, which also possessed the higher affinity (*K*_a_) value. In contrast, [82,83] did not exhibit statistical differences among the calculated values for flavonoids and proanthocyanidins, respectively, against PL activity, while Li et al. [65] found a statistical difference between the *K*_sv_ values from the best and worst inhibitors, quercetin and apigenin, respectively, of TP activity. If the size of BSA and TP are compared with the size of PL, this may indicate that the molecular weight of the protein, could influence the efficiency of the quencher [68]. Finally, the *k*_q_ value for PCs–enzyme interactions is calculated to confirm the quenching mechanism of the ground-state complex formation (static), when its value is higher than the maximum scatter collision quenching constant for quenchers [82,98].

**Figure 4:**
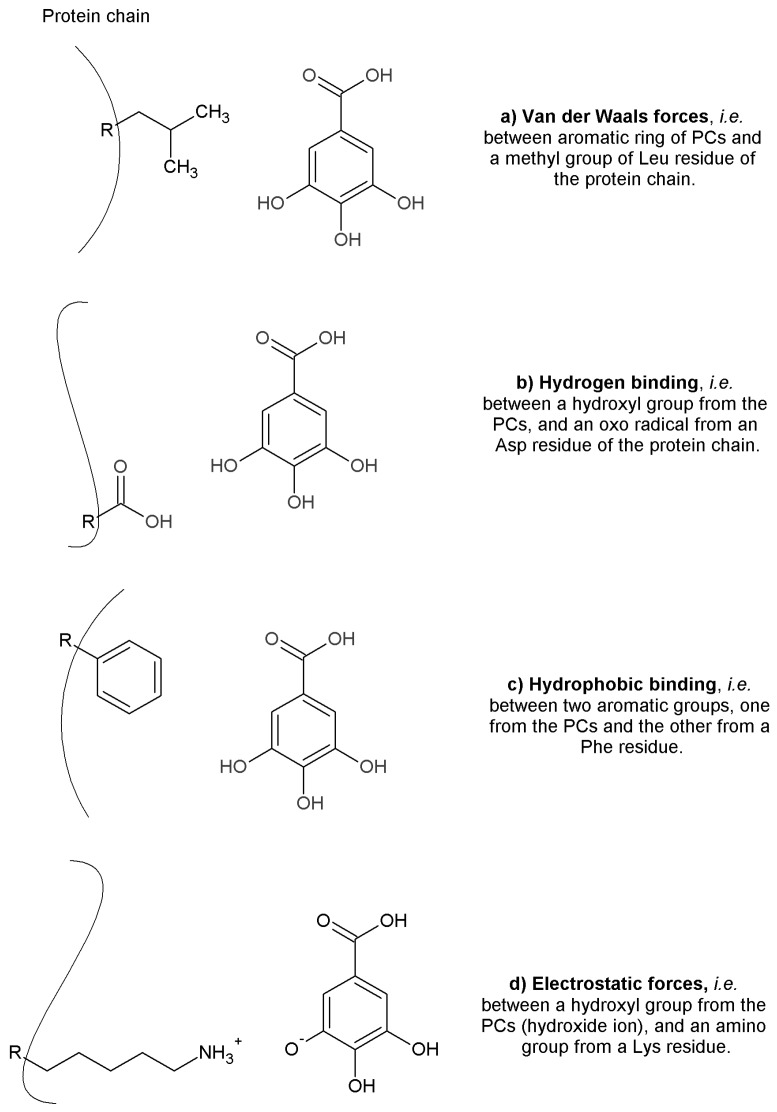
Non-covalent binding involves in the PCs–enzymes interactions. Examples of (**a**) van der Waals forces; (**b**) hydrogen binding; (**c**) hydrophobic binding; and (**d**) electrostatic forces. The protein chain is represented by *R* and curved line.

Thermodynamic parameters, such as enthalpy change (Δ*H*), entropy change (Δ*S*), and Gibbs free energy change (Δ*G*) values have been calculated by fluorescence spectroscopy, at different temperatures, for the interaction between PCs and digestive enzymes [29,66,83]. Assuming that Δ*H* is constant, the calculated binding constants at different temperatures, are used to assess the Δ*G* and Δ*S* values trough the van’t Hoff Equation [64]. These thermodynamic parameters provide information on the type of main non-covalent interactions present in the PCs–enzyme system [83], which can be driving by hydrophobic binding, electrostatic forces, and van der Waals forces mainly as hydrogen binding [101] (Figure 4). According to Ross and Subramanian [102], if both Δ*H* and Δ*S* are positive, hydrophobic binding are the main interactions present in the system, as Wu et al. [64] found for the interactions between PCs glycosides extracted from tea (*Ligustrum purpurascens*) and the proteases, PE, TP and CT. Negative Δ*H* and Δ*S* values suggest that van der Waals forces were the main forces that describe that system. Moreover, a positive Δ*S* value accompanied with a negative Δ*H* value for a system, reveal that electrostatic forces mostly occur as Wu et al. [11] calculated for PL-acteoside system. All the authors have observed a Δ*G* negative value, which indicated that their respective studied interactions were spontaneous. It is a statement that a negative Δ*G* value favored association. If it is matter of study the PCs–enzyme interaction, it is important to mentioned Δ*G* values to compared them. There no exists a complete convention yet to stablish which negative Δ*G* value (or rank) corresponds to each type of interaction. In other words, a pattern in the magnitude of Δ*G* values for PCs–enzyme interactions is not clear enough. Similarly, Xiao et al. [29] estimated that the main forces driving the interaction between tannic acid, and TP and AG were hydrophobic binding (positive Δ*H* and Δ*S*) and the complex formation was spontaneous (negative Δ*G*) in both cases. Two conclusions are obtained, first, all the reactions between PCs and digestive enzymes were spontaneous (static quenching results). Also, when lower molecular weight PCs interacted with the enzymes, van der Waals forces and hydrogen binding were observed, i.e., by Li et al. [83] with flavonoids and PL, and Narita and Inouye [69] with chlorogenic acids derivatives and PA; while other electrostatic forces and hydrophobic binding were determined for higher molecular weight PCs as acteoside with PL [11], or tannic acid with AG and TP [29]. 

As Figure 4 shows, the partially charged and charged groups in the enzyme are generally linked by van der Waals forces, hydrogen binding and other electrostatic forces with similar groups in PCs [11,29,64]. Van der Waals forces might occur between aromatic ring from PCs and a methyl group from a Leu or Val residue of the enzyme [102]. Hydrogen binding might also occur between two hydroxyl groups from two aromatic rings, one from the PCs, and the other from an aromatic residue in the enzyme [28,64,68]. This has been pointed out for the interaction between PA and acarbose related compounds [26]. Hydrophobic binding can occur between two aromatic rings (*pi*-stacking interaction), one from an hydrophobic amino acid (i.e., Phe) of the enzyme, and the other from the PCs structure [101].

Isothermal titration calorimetry (ITC) is used to accurately determine the thermodynamic parameters of PCs-binding (Δ*H*, Δ*S*, Δ*G* and *n*). There are just a few studies using this technique. For example, Wu et al. [11] applied ITC to analyze the acteoside-PL interaction; Trivella et al. [103] used for other PCs–proteins systems such as flavonoids (quercetin, naringenin, and others) and transthyretin, a carrier protein involved in human amyloidosis; and Budryn et al. [104] studied the interaction of phenolic acids (caffeic, ferulic, and others) and whey, egg white and soy protein isolates. Another barely used method for PCs–digestive enzymes interactions is the differential scanning calorimetry (DSC), where the transition midpoint temperature (*T*_m_) of PCs–digestive enzymes complexes is calculated [82]. Since the *T*_m_ values analysis, Wang et al. [82] deduced that proanthocyanidins led to enhancement of the thermostability of PA. Circular dichroism (CD) has been also used to determine changes induced by PCs binding to the protein structure. CD results provide information on the enzyme secondary elements, such as percentages of α-helix, β-sheet, turns, and random (or unordered) coil. CD studies of the complexes between PCs and PL, PE, TP, and CT showed a decrease in the α-helical structures, as the PCs–enzyme ratio increased (up to 1:2) [11,64]. Wu et al. [11] observed decreases from 35% to 30%, whereas Wu et al. [64] observed decreases of around 1%–2% in complexes formed between different PCs and proteases (PE, TP and CT). Wu et al. [64] also found a decrease of β-sheet structures (losses between 1.6% and 3.2%) for the same PCs–protease complexes. Carbohydrate–hydrolyzing enzymes, such as PA, mostly conformed by β-sheet structures, did not exhibit changes on its α-helix structures [68]. An increase of turns structures as β-sheet structures decrease was also observed [14,68]. All these changes on the secondary structure (α-helix, β-sheet, and turns) of the proteins in the presence of PCs were increasing the percentage of random coil, up to 7% (from 47.5% to 54.3%) for PE. CD results suggest that the PCs–enzyme interaction may cause a conformational change, and the subsequent unfolding of the protein structure, which lead to a decrease of the enzymatic activity [11,64,68,82]. However, due to the scarcity of CD studies of PCs–digestive enzymes complexes there are not sufficient studies for a complete SAR analysis, that explains the changes on the protein conformation depending on the protein and PCs structures. 

### 2.4. Mechanisms of Enzymatic Inhibition

Table 2 describes the kinetic parameters obtained for the different PCs–digestive enzymes systems, using both Michaelis–Menten (M–M) and Lineweaver–Burk (L–B) analysis. The main inhibition mode determined for PCs–digestive enzymes is non-competitive [11,25,64,77,82,83]. Wang et al. [82] observed a decrease in *V_max_* values for PL, when grape proanthocyanidins were added, without a significant change in *K*_M_ value, suggesting that the affinity of enzyme for substrate was not affected and a non-competitive inhibition occurred. Shobana et al. [77] analyzed the interaction between finger millet (*Eleusine coracana* L.) PCs extract and PA, observing that *V_max_* value changed depending on the PCs concentration. Li et al. [83] reported that binding to enzyme–substrate (ES) complex occurred when Tartary buckwheat bran flavonoids interacted with PL, indicating a non-competitive type inhibition. The results of a non-competitive inhibition were supported by analysis such as FLU, ITC and CD analysis, as Wu et al. [11] reported with PL-acteoside system. 

A mixed-type inhibition of AG and TP activity by tannic acid has been reported [29]. This type of inhibition was also reported by da Silva et al. [12] for the inhibition of PA by pinhão (*Araucaria angustifolia*) PCs extract, rich in condensed tannins. Narita and Inouye [69] observed a mixed-type inhibition for all the chlorogenic acids derivatives tested over the PA activity, and, considering the *K*_i_ and *K*_i_’ values, they suggested that these PCs present higher affinity for the ES complex. Also, lower values of these constants were correlated to the presence of more chlorogenic acid sub-structures (moieties). This higher affinity to the ES complex is in agreement with those results reported for the interaction of Tartary buckwheat flavonoids with PL [83]. 

For some specific PCs, not extracts, competitive type inhibition was observed. Quercetin showed a competitive type inhibition (unchanging *V_max_*) when interacting with AG [67]. Hu et al. [81] reported that caffeoylquinic acid and isomers showed a competitive type inhibition over PL, and the same mechanism was observed with twelve flavonoids isolated from *Glycyrrhiza glabra* roots [85]. Cai et al. [68] evaluated the competitive type inhibition of PA activity by sorghum procyanidins. The authors also pointed out that the one binding site predicted by fluorescence spectroscopy would be located in the vicinity of the active site, in agreement with their molecular docking analysis (MOD). MOD is one of the alternative tools that can be applied to represent an image of the possible sites were the PCs can bind the protein, by predicting the binding preferences. In Table 1, we can observe that a few authors performed virtual screening by MOD to support their in vitro results [11,64,66,81,85], while other authors pointed out a possible ligand binding site without this analysis [68,69] as Table 2 exhibits. In a MOD study, the prediction of PCs binding sites requires to consider aspects as the characteristics of the ligand (PCs), in order to obtain the minimal free energy change (minimized energy building) and the structure of ligand-protein complex [85], and the most favorable binding pockets and ligand conformations are proposed [81]. Zeng et al. [66] studied by MOD the interaction of the flavonoid baicalein with PE, observing that the flavonoid interacted with the hydrophobic cavity of the enzyme, in agreement with their fluorescence quenching data. Wu et al. [11] suggested, through MOD analysis, that the observed non-competitive inhibition of acteoside over PL was produced by an alteration of the enzyme molecular conformation which reduced its catalytic activity. They suggested that hydrogen bonding were the main interactions occurring between acteoside and PL. Hu et al. [81] and Birari et al. [85] also supported their kinetic results of a competitive inhibition with MOD analysis. Hu et al. [81] used MOD analysis to describe their competitive inhibition model as a consequence of the binding of caffeoylquinic acid isomers to residues from the catalytic triad of PL (His^263^, Asp^176^ and Ser^152^), in agreement with Kokotos [105] who described a similar interaction between a ligand and Ser^152^ of human PL. In contrast, Wu et al. [64] suggested that the hydrogen binding of acteoside to one amino acid residue of the catalytic triad of PE (Asp^32^) and CT (Ser^195^) might not be associated to a competitive type inhibition. The authors mentioned that the number and strength of hydrogen binding between amino acid residues and PCs have to be carefully considered, because the strongest binding affinity and the highest inhibitory activity are directly related to them [81]. The use of different and innovative modeling software, such as molecular dynamic simulation would provide information about the possible flexibility of PCs–enzyme complexes and their stability, by truncation fitting to experimental data [106,107]. The truncation step means to eliminate, in silico, one or a few amino acids from the protein, and compare the binding of ligands with both enzymes (native and truncated) structures [106].

### 2.5. Conditions Influence the Binding of Polyphenolic Compounds with Digestive Enzymes

Reaction conditions such as pH and temperature can modify the binding and interactions between PCs and proteins [68,95,97,108], including digestive enzymes [83]. Studies between PE and tannins [97] have shown that the solubility (or hydrophilicity) of PCs might decrease if the experimental pH or temperature decrease, and consequently their interactions with the enzymes will be modified. Li et al. [83] observed that an increase of temperature did not accelerate molecular diffusion of protein fluorophore (*K*_sv_ value would increase with the increase of temperature) to collide with PCs. Also, the affinity and the binding sites (*K*_a_ and *n* values, respectively) of PL and flavonoids, quercetin, isoquercetin and rutin, decreased as the temperature increased (up to 32 °C). Similar results were observed by the interaction of PCs and proteins, examples such as the interaction of (−)-epigallocatechin with bovine β-lactoglobulin, where the increasing temperature (up to 40 °C) affected the binding and the stability of the system [94]. The authors mentioned that the increasing temperature may result in the increasing diffusion coefficient which corresponds to a collisional quenching mechanism, but it also represented instability of this system. Prigent et al. [95] observed that the affinity between a chlorogenic acid derivative (5-CQA) and BSA decreased with increasing temperature (up to 60 °C), while no significant effect was observed due to pH or ionic strength changes. For some non-digestive enzymes, it was observed experimental pH (from 2 to 6) affected the interaction with catechin and tannic acid, especially at pH values above the isoelectric point of the protein solution [108]. 

Probably the lack of analysis at various pH conditions of the interaction between PCs and digestive enzymes is due to the knowledge of the actual conditions in which these interactions take place in in vivo or in vitro the digestive systems, which have been analyzed and reviewed by several authors [109,110].

## 3. Conclusions

Commonly employed in vitro techniques can provide excellent data regarding PCs–enzyme interactions, but it seems that novel in silico approaches will be the next step to complete these studies, before in vivo assessments. The non-covalent interactions are the key of the enzymatic inhibition, because these interactions are the basis of reversible inhibitions that may result potentially convenient for certain medical therapies. A higher binding and inhibitory ability of PCs with these digestive enzymes is mainly related to the PCs structure, and binding characteristics will influence the catalytic activity of the enzymes. The characteristics of PCs to consider are the number of hydroxyl groups, and their location at the B and C rings; the presence of glycosylation on the PCs, the position and number of glycosyl units, and the structural complexity of the PCs or number of PCs moieties (for example in the polymeric and oligomeric proanthocyanidis). In some instances, the presence of an extra single phenolic hydroxyl group can modify the effect of the PCs on the catalysis. Other aspects that influence the PCs–enzyme interaction are the composition of the enzyme (i.e., its number of polar and hydrophobic amino acids, its molecular weight), and characteristics of the reaction (pH, temperature, and time of incubation). In this way, the better understanding of the mechanisms in which natural inhibitors such as PCs act upon the digestive enzymes would allow to find alternatives to the current commercial inhibitors. The possible benefit of the inhibition of digestive enzymes activities should have to be made clear, in order to be able of recognize the effect on the efficacy of PCs. 

## Figures and Tables

**Table 1:** In vitro studies between PCs and digestives enzymes from porcine source.

Enzyme	PCs Extract	PCs Identified on the Extracts by HPLC	PCs Standard	Assessment Methods										Note	Reference
				UVV			FLU		ITC	DSC	CD	MOD	Others		
				% Inh	IC_50_	KIN	AFF	THR							
**AG**	ND	ND	Tannic acid	*	*	*	*	*						KIN calculated parameters (*K_M_* and *V_max_*); THR calculated parameters (Δ*G*, Δ*H* and Δ*S*); and AFF calculated parameters: *K*_sv_, *k*_q_, *K*_a_, *n*.	[29]
	Twenty Canadian lentil cultivars (*Lens culinaris*)	Twenty one mainly: *p*-hydroxybenzoic acid, syringic acid, epicatechin gallate, quercetin-3-xyloside, quercetin-3-glucoside, kaempferol-3-glucoside	Catechin, epicatechin, procyanidin B1, kaempferol, kaempferol-glucose, quercetin, quercetin-arabinose		*										[21]
	Australian fruits (*Davidson pruriens* and *Santalum acuminatum*)	Ellagic acid, flavonoids (myricetin, quercetin, rutin), ellagitannins, and anthocyanins	ND		*										[27]
	Muscadine (*Vitis rotundifolia*)	Twelve like catechin, ellagic acid, quercetin	ND		*	*								KIN including *K*_i_.	[67]
	Tasmannian pepper (*Drymis lanceolata*) leaf, anise myrtle (*Syzygium anisatum*), lemon myrtle (*Backhousia citriodora*)	Ellagic acid, chlorogenic acid, flavonoids (catechin, myricetin, hesperetin, quercetin)	ND	*	*										[61]
**PA**	Green, black and oolong tea (*Camellia sinensis*)	ND	ND	*	*										[25]
	Sorghum	Procyanidins	ND				*				*			AFF (*K*_sv_, *k*_q_, *K*_a_).	[68]
	Pinhão coat (*Araucaria angustifolia* and *A. mearnsii*)	Condensed tannin	ND			*							*	KIN parameters; Measurement of post-prandial glycemic levels in healthy rats.	[12]
	Black chokeberry (*Aronia melanocarpa* L.)	Phenolic acids as chlorogenic acid, and anthocyanins as cyanidin-3-glucoside	ND		*										[62]
	Green coffee (*Coffea arabica*) beans	Eight chlorogenic acids derivatives: three subgroups of caffeoylquinic acids, feruloylquinic acids, and dicaffeoylquinic acids	ND		*	*								KIN parameters.	[69]
	Cumin (*Cuminum cyminum* L.)	ND	ND	*									*		[70]
	Nepalese medicinal herb Pakhanbhed (*Bergenia ciliata*)	(-)-3-*o*-galloylepicatechin and (-)-3-*o*-galloylcatechin	ND		*										[37]
	Chinese green tea	Catechin	ND	*	*	*								KIN parameters.	[28]
	Strawberry (*Fragaria vesca* L.)	PCs fractions	ND												[71]
**AG, PA**	Quinoa (*Chenopodium quinoa*)	Phenolic, flavonoid, and condensed tannins contents	ND	*	*										[72]
	Five species of *Myrcia* genus	Gallic acid, flavan-3-ols, and flavonols	ND	*	*										[73]
	Chañar (*Geoffrea decorticans*) fruit	Caffeic acid, protocatechuic acid, vanillic acid, *p*-coumaric acid among others	Quercetin		*										[22]
	Soybean (*Glycine max*)	Bound and free phenolic extract	ND		*										[74]
	*Nelumbo nucifera* leaves	Flavonoids	ND		*								*	Measurement of total cholesterol, triacylglyceride and low-density lipoprotein cholesterol contents in high fat diet-fed rats, and others.	[75]
	Six herbal teas	From *Plantago lanceolata*, L.: chlorogenic acid, rutin, among others	Gallic acid, catechin, among others	*	*										[58]
	Algae (*Palmaria, Ascophyllum* and *Alaria*)	Tannins	ND		*										[76]
	Guava (*Psidium guajava* L.) leaves	Quercetin, kaempferol, guaijaverin, avicularin, myricetin, hyperin, and apigenin.	ND	*	*										[60]
	Finger millet (*Eleusine coracana* L.) seed	Gallic acid, caffeic acid, kaempferol, among others.	ND		*	*								KIN parameters.	[77]
	ND	ND	Flavonoids	*	*										[78]
	Black soybean (*Glycine max*) and black turtle beans (*Phaseolus vulgaris*)	ND	Phenolic acids (gallic acid, syringic acid and others), and flavonoids (catechin, quercetin-3-*o*-glucoside)	*	*										[79]
**PL**	Peanut (*Arachis hypogaea* L.)	ND	ND	*									*	Measurement of body weight, liver size, fecal lipid excretion and triacylglyceride content in high fat diet-fed rats.	[80]
	Chañar fruit	Caffeic acid, protocatechuic acid, vanillic acid, *p*-coumaric acid among others.	Quercetin		*										[22]
	Twenty Canadian lentil cultivars	Twenty one as: *p*-hydroxybenzoic acid, and quercetin-3-*o*-glucoside.	Quercetin and quercetin-arabinoside		*										[21]
	ND	ND	Cinnamic acid		*								*	Measurement of body weight, total cholesterol and triacylglyceride contents in high fat diet-fed rats, and others.	[20]
	ND	ND	3-caffeoylquinic acid (CQA), 4,5-CQA, 3,4-CQA, 3,5-CQA, and 4,5-diCQA		*							*			[81]
	Twenty eight traditional Thai medicinal herbs	ND	ND	*	*										[57]
	Horseradish (*Armoracia rusticana*)	ND	ND		*										[6]
	ND	ND	Acteoside	*			*		*		*	*			[11]
	ND	ND	Proanthocyani-dins	*		*	*			*	*		*	KIN parameters; AFF parameters: *K*_sv_, *k*_q_, *K*_a_, *fa*; and formation of protein aggregates. Also a hydrodynamic radius analysis was performed.	[82]
	Black chokeberry	Phenolic acids and anthocyanins	ND		*										[62]
	Australian fruits	Ellagic acid, ellagitannins, flavonoids and anthocyanins	ND	*	*										[27]
	*Nelumbo nucifera* leaves	Flavonoids	ND		*									Measurement of lipid components such as triacylglyceride, total cholesterol, and others in high fat diet-fed rats.	[75]
	Six herbal teas	From *Plantago lanceolata*, L.: chlorogenic acid, rutin, among others	Gallic acid, catechin, among others.	*	*										[58]
	Muscadine	Twelve like catechin, ellagic acid, and quercetin	ND		*	*								KIN parameters and *K*_i_.	[67]
	Green tea and grape seeds	ND	Epigallocatechin-3-gallate, kaempferol, and quercetin	*	*										[34]
	Tasmannian pepper leaf, anise myrtle, lemon myrtle	Ellagic acid, chlorogenic acid, and flavonoids (i.e., catechin)	ND	*	*										[61]
	Tartary buckwheat bran	ND	Quercetin, isoquercetin and rutin		*	*	*	*							[83]
	Black tea	Polymerized polyphenol fraction	Polymerized catechins such as theaflavin and theaflavin-3-gallate		*								*	Measurement of triacylglyceride content and body weight in high fat diet-fed rats.	[84]
	Root of *Glycyrrhiza glabra*	Twelve flavonoids	ND		*							*	*	Measurement of body weight, total cholesterol and triacylglyceride contents in high fat diet-fed rats.	[85]
	White and green tea	Flavan-3-ols for green tea, and 5-galloyl quinic acid, digalloyl glucose, trigalloyl glucose and strictinin for white tea	ND	*	*										[86]
	Berries (blackcurrantrowan, blueberry, lingonberry, among others)	Tannins: ellagitannin and proanthocyanidin	ND	*	*										[18]
	Cumin	ND	ND	*											[70]
	Chinese green tea	Catechin	ND	*											[28]
	Peanut	ND	ND	*									*	Measurement of body weight, liver size, fecal lipid excretion and triacylglyceride content in high fat diet-fed rats.	[80]
	Seventy five medicinal plants	ND	ND	*									*	A radioactive method was used.	[87]
	Black soybean (*Glycine max*) and black turtle beans (*Phaseolus vulgaris*)		Phenolic acids (gallic acid, syringic acid and others), and flavonoids (catechin, quercetin-3-*o*-glucoside)	*	*										[79]
**PE**	ND	ND	Ten flavonoids (principally baicalein)		*		*	*				*		AFF pameters: *K*_sv_, *k*_q_.	[66]
**TP**	ND	ND	Tannic acid	*	*	*	*							AFF parameters: *K*_sv_, *k*_q_, *K*_a_, *n*.	[29]
	ND	ND	Flavonoids as quercetin, luteolin, and kaempferol	*			*							AFF parameters: *K*_sv_, *k*_q_.	[65]
	ND	ND	Gallic acid	*											[88]
	ND	ND	Procyanidins (catechin)		*	*	*						*	Dynamic light scattering and nephelometry were used too.	[89]
**PE, TP**	Chinese green tea	Catechin	ND	*											[28]
**PE, TP, CT**	Tea (*Ligustrum purpurascens*)	Phenylpropanoid glycosides (like acteoside)	ND	*			*	*			*	*		AFF parameters: *K*_a _and *n*.	[64]

PE was included in the table, due to the lack of PCs–proteases interaction studies. Abbreviations: PCs, Polyphenolic compounds; AG, α-Glucosidase; PA, Pancreatic alpha-amylase; PL, Pancreatic lipase; PE, Pepsin; TP, Trypsin; CT, Chymotrypsin; % Inh, % Inhibition; IC_50_, concentration required to inhibit enzyme activity by 50%; UVV, UV–Vis spectroscopy; FLU, Fluorescence spectroscopy; KIN, kinetic; AFF, affinity; THR, thermodynamic; ITC, Isothermal titration calorimetry; DSC, Differential scanning calorimetry; CD, Circular dichroism; MOD, Molecular docking; *K_M_*, Michaelis-Menten constant; *V_max_*, maximal velocity; *K*_i_, inhibition constant; *K*_sv_, collisional quenching constant; *K*_a_, affinity constant; *k*_q_, quenching rate constant (bimolecular quenching constant); *n*, binding sites; *fa*, fraction of fluorophore accessible to the quencher; ND, No data. * Analysis were determined by the corresponding method.

**Table 2:** Characteristics of the in vitro interaction between PCs and porcine digestive enzymes.

Enzyme	PCs	Inhibition Type	Type of Binding or Force	Binding Site in the Enzyme	Reference
AG	Tannic acid	Mixed-type	Hydrophobic and electrostatic	ND	[29]
	Twelve PCs such as catechin, ellagic acid, and quercetin	Competitive	ND	ND	[67]
PA	Teas (green, black and oolong tea)	Non-competitive	ND	ND	[25]
	Procyanidins	ND	Hydrophobic	One site	[68]
	Condensed tannins	Mixed-type	ND	ND	[12]
	Eight Chlorogenic acids: three subgroups of caffeoylquinic acids, feruloylquinic acids, and dicaffeoylquinic acids	Mixed-type	Hydrogen	Non-catalytic sites	[69]
	Catechin	ND	Hydrogen and hydrophobic	ND	[28]
AG, PA	Gallic acid, caffeic acid, kaempferol, and others	Non-competitive	ND	ND	[77]
PL	3-caffeoylquinic acid (CQA) derivatives as 4,5-CQA, 3,4-CQA, 3,5-CQA, and 4,5-diCQA	Competitive	Hydrogen and hydrophobic	Catalytic triad	[81]
	Acteoside	Non-competitive	Hydrogen	Non-catalytic sites	[11]
	Proanthocyanidins	Non-competitive	Weak	ND	[82]
	12 like catechin, ellagic acid, as quercetin	Competitive	ND	ND	[67]
	Quercetin, isoquercetin and rutin	Non-competitive	Hydrophobic and van der Waals	One site	[83]
	12 flavonoids such as isoliquiritigenin	Competitive	Hydrogen	Catalytic site	[85]
	Catechin	ND	Hydrogen and hydrophobic	ND	[28]
PE	10 Flavonoids (principally baicalein)	ND	Hydrophobic and electrostatic	One hydrophobic site or cavity	[66]
TP	Tannic acid	Mixed-type	Hydrophobic and electrostatic	ND	[29]
	Gallic acid	ND	Hydrophobic	ND	[88]
	Procyanidins (catechin)	Competitive	Hydrogen bonds	Near to catalytic site	[99]
PE, TP	Catechin	ND	Hydrogen and hydrophobic	ND	[28]
PE, TP, CT	Phenylpropanoid glycosides like acteoside	Non-competitive	Hydrogen, hydrophobic, van der Waals and electrostatic	Catalytic sites	[64]

PE was included in the table, due to the lack of PCs-–-proteases interaction studies. Abbreviations: PCs, Polyphenolic compounds; AG, α-Glucosidase; PA, Pancreatic alpha-amylase; PL, Pancreatic lipase; PE, Pepsin; TP; Trypsin; CT, Chymotrypsin; ND, No data.
